# The clinical role of microRNA-21 as a promising biomarker in the diagnosis and prognosis of colorectal cancer: a systematic review and meta-analysis

**DOI:** 10.18632/oncotarget.16488

**Published:** 2017-03-22

**Authors:** Qiliang Peng, Xueli Zhang, Ming Min, Li Zou, Peipei Shen, Yaqun Zhu

**Affiliations:** ^1^ Department of Radiotherapy & Oncology, Second Affiliated Hospital of Soochow University, Suzhou, China; ^2^ Institute of Radiotherapy & Oncology, Soochow University, Suzhou, China; ^3^ Suzhou Key Laboratory for Radiation Oncology, Suzhou, China; ^4^ Center for Systems Biology, Soochow University, Suzhou, China; ^5^ School of Medicine, Örebro University, Örebro, Sweden

**Keywords:** miR-21, diagnosis, prognosis, colorectal cancer, meta-analysis

## Abstract

This systematic analysis aimed to investigate the value of microRNA-21 (miR-21) in colorectal cancer for multiple purposes, including diagnosis and prognosis, as well as its predictive power in combination biomarkers. Fifty-seven eligible studies were included in our meta-analysis, including 25 studies for diagnostic meta-analysis and 32 for prognostic meta-analysis. For the diagnostic meta-analysis of miR-21 alone, the overall pooled results for sensitivity, specificity, and area under the curve (AUC) were 0.64 (95% CI: 0.53-0.74), 0.85 (0.79-0.90), and 0.85 (0.81-0.87), respectively. Circulating samples presented corresponding values of 0.72 (0.63-0.79), 0.84 (0.78-0.89), and 0.86 (0.83-0.89), respectively. For the diagnostic meta-analysis of miR-21-related combination biomarkers, the above three parameters were 0.79 (0.69-0.86), 0.79 (0.68-0.87), and 0.86 (0.83-0.89), respectively. Notably, subgroup analysis suggested that miRNA combination markers in circulation exhibited high predictive power, with sensitivity of 0.85 (0.70-0.93), specificity of 0.86 (0.77-0.92), and AUC of 0.92 (0.89-0.94). For the prognostic meta-analysis, patients with higher expression of miR-21 had significant shorter disease-free survival [DFS; pooled hazard ratio (HR): 1.60; 95% CI: 1.20-2.15] and overall survival (OS; 1.54; 1.27-1.86). The combined HR in tissues for DFS and OS were 1.76 (1.31-2.36) and 1.58 (1.30-1.93), respectively. Our comprehensive systematic review revealed that circulating miR-21 may be suitable as a diagnostic biomarker, while tissue miR-21 could be a prognostic marker for colorectal cancer. In addition, miRNA combination biomarkers may provide a new approach for clinical application.

## INTRODUCTION

Colorectal cancer (CRC) is one of the most commonly diagnosed malignancies and a major cause of cancer-associated mortality all over the world [1]. The most reliable procedure for diagnosis is mainly based on colonoscopy, which is invasive and unpleasant for patients to undergo [2]. There is thus an urgent need for a noninvasive biomarker that can detect CRC with high precision. In addition, the mortality of CRC remains high due to the late diagnosis or lack of an effective therapeutic option [3, 4]. No effective prognostic molecular marker that can predict the clinical outcome and then provide guidance for treatment selection has been developed [5]. Consequently, there is a great need to explore new efficient methods for CRC diagnosis along with prognosis.

MicroRNAs (miRNAs) are small, endogenous, noncoding RNAs that can regulate the expression of genes at the post-transcriptional level [6]. Increasing evidence has demonstrated that miRNAs play vital roles in multiple biological processes, such as cell growth, differentiation, apoptosis, invasion, and metastasis [7]. Given their critical involvement in the development of tumors via oncogenic or tumor-suppressive properties, altered profiles of miRNAs have been shown to be related to carcinogenesis and tumor progression [8]. Hence, miRNAs may be useful biomarkers in the early detection of cancer and for predicting therapeutic efficacy as well as prognosis [9, 10].

miR-21 stands out as the most representative miRNA biomarker as it has been extensively explored in a range of studies on numerous cancers [11]. Considerable research has been conducted on the use of miR-21 expression to distinguish between CRC patients and normal controls for a range of different sample sourceses, suggesting the great promise of miR-21 as a novel biomarker in screening CRC. Meanwhile, substantial evidence has also revealed that miR-21 might be a useful predictor of the clinical outcome as its expression level is significantly related to the prognosis of CRC patients.

However, the results of studies on miR-21 reported to date remain inconclusive, which may be due to small sample sizes, different disease statuses, different sample sources, different detection methods, and other uncontrolled factors. Although several published meta-analyses have been conducted in response to these conflicting results, there are still limitations to the obtained findings. Most meta-analyses merely focused on the utility of miR-21 as a potential marker in the diagnosis or prognosis of CRC [12–16]. In addition, they separately investigated the value of diagnosis or prognosis in serum, plasma, or feces [12, 13, 15, 17]. Furthermore, some of them were conducted using a relatively small number of studies [12, 14–18]. Moreover, ignoring the heterogenicity in different microRNAs or different cancer types, many researchers merged miR-21 with various other microRNAs to draw conclusions on the value of all microRNAs in CRC [19–22], while some investigators included studies of miR-21 for CRC along with studies for various other cancers to determine the value of miR-21 in cancer [17, 23–28], which discussed too little in our concerned topic. Finally, most of the published meta-analyses focused on the miR-21 biomarker alone [12, 13, 15–18], although systematic meta-analyses that evaluate combinations of biomarkers may provide more useful information about the potential value of future biomarkers. Combined biomarkers or combination biomarkers, which are combinations of several markers, have been shown to improve the prediction accuracy compared with a single biomarker [29, 30] and were thus identified as having great potential for the diagnosis or prognosis of CRC.

Considering the limits of existing publications, we conducted a more integrative meta-analysis of miR-21 for CRC based on all relevant reported studies to obtain a better understanding of the diagnostic and prognostic efficiency of miR-21 in CRC. Furthermore, by focusing not only on a single biomarker, we discussed whether combination markers are more effective than individual ones.

## RESULTS

### Selection of studies

**Figure 1:**
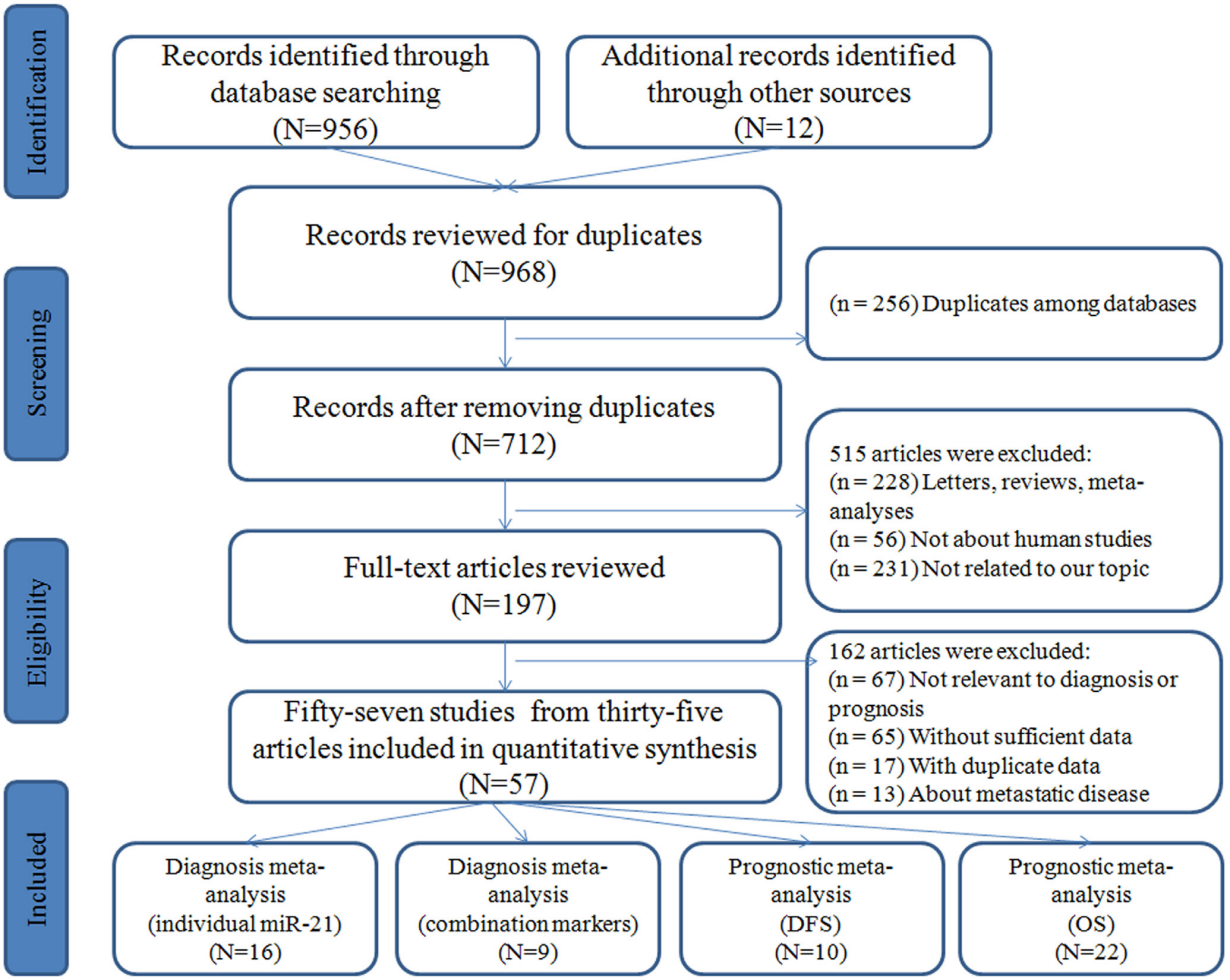
Flow diagram of the study selection process

The flow diagram for the literature search is presented in Figure 1. The initial search from the selected literature databases and other sources returned a total of 968 articles. After careful exclusion of inappropriate ones in each step, 57 published studies were finally included in this meta-analysis, including 25 studies for diagnostic meta-analysis and 32 for prognostic meta-analysis. Among the studies for diagnosis, 16 studies were about miR-21 alone [18, 31–42] and 9 were about miR-21-related combination markers [31, 32, 35, 36, 43, 44]. With respect to prognosis, 10 studies were connected with disease-free survival (DFS) [45–51] and 22 were related to overall survival (OS) [18, 36, 37, 45–47, 49–59].

### Diagnostic meta-analysis

### Diagnostic meta-analysis of miR-21 alone in CRC

#### Study characteristics and quality assessment

A total of 15 publications on 16 studies involving 1270 cases and 944 controls were analyzed. The main features of all of the 16 included studies are shown in Table 1A. The expression of miR-21 was detected using quantitative real-time reverse transcription PCR (qRT-PCR) in all studies. A total of 11 studies were performed on Asian populations and 5 on Caucasian ones. Sample sources were classified as serum (*n* = 5), plasma (*n* = 6), feces (*n* = 4), and tissue (*n* = 1). Assessments of the quality of these studies are also given in Table 1A, suggesting that overall they were of moderate to high quality.

**Table 1A:** The main features of the included studies on individual miR-21

Author	Year	Country	Ethnicity	Case/Control	Sample	AUC	Se	Sp	QUADAS
Koga et al	2010	Japan	Asian	197/119	Feces	Na	14.7%	91.6%	4
Wu et al	2010	China	Asian	27/48	Feces	Na	50.0%	83.0%	4
Wu et al	2012	China	Asian	88/101	Feces	0.64	55.7%	73.3%	4
Kanaan et al	2012	America	Caucasian	30/30	Plasma	0.820	90.0%	90.0%	4
Kanaan et al	2012	America	Caucasian	20/20	Plasma	0.910	81.0%	94.0%	4
Wang et al	2012	China	Asian	32/39	Serum	0.85	87.5%	74.4%	5
Kuriyama et al	2012	Japan	Asian	138/126	Feces	0.80	39.0%	97.6%	6
Luo et al	2013	Germany	Caucasian	80/144	Plasma	0.653	51.7%	80.7%	3
Liu et al	2013	China	Asian	200/80	Serum	0.802	65.0%	85.0%	6
Toiyama et al	2013	Japan	Asian	186/53	Serum	0.927	82.8%	90.6%	5
Kawata et al	2014	Japan	Asian	88/11	Serum	0.798	61.4%	90.9%	5
Zhang et al	2014	China	Asian	41/30	Plasma	0.657	51.2%	79.0%	6
Zanutto et al	2014	Italy	Caucasian	29/29	Plasma	0.647	58.0%	58.0%	5
Basati et al	2014	Iran	Asian	40/40	Serum	0.87	77.0%	78.0%	5
Omrane et al	2014	France	Caucasian	25/25	Tissue	0.746	68.0%	72.0%	4
Du et al	2014	China	Asian	49/49	Plasma	0.877	76.2%	93.2%	5

#### Diagnostic accuracy of l miR-21 alone in CRC

**Figure 2:**
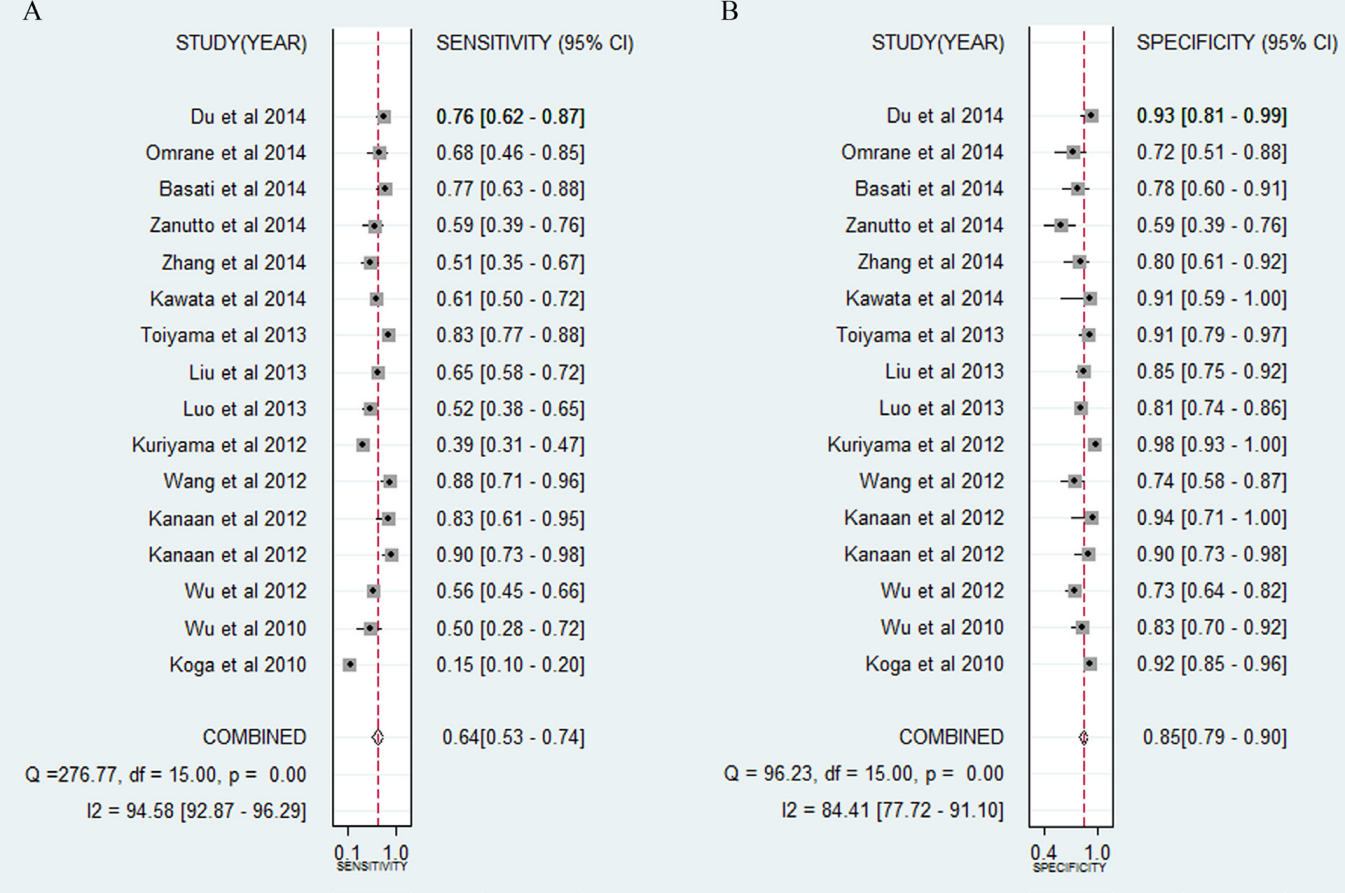
Forest plots of sensitivities and specificities of individual miR-21 in the diagnosis of CRC

Forest plots for the enrolled studies on the pooled sensitivity and specificity are shown in Figure 2. Significant heterogeneity was observed between studies for the high I^2^ values in sensitivity (94.58%) and specificity (84.41%). The overall combined sensitivity, specificity, and the diagnostic odds ratio (DOR) were 0.64 (95% CI: 0.53-0.74), 0.85 (0.79-0.90), and 10.33 (5.85-18.24), respectively. The DOR value meant that someone who was found to be positive for CRC with a high level of miR-21 had a 10.33-fold higher chance ofactually sufferring from CRC compared with someone with a negative CRC result. The summary receiver operator characteristic (SROC) curve (Figure 4A) was plotted and the corresponding area under the SROC curve (AUC) was calculated to be 0.85 (0.81-0.87), revealing moderate diagnostic accuracy overall.

**Figure 3:**
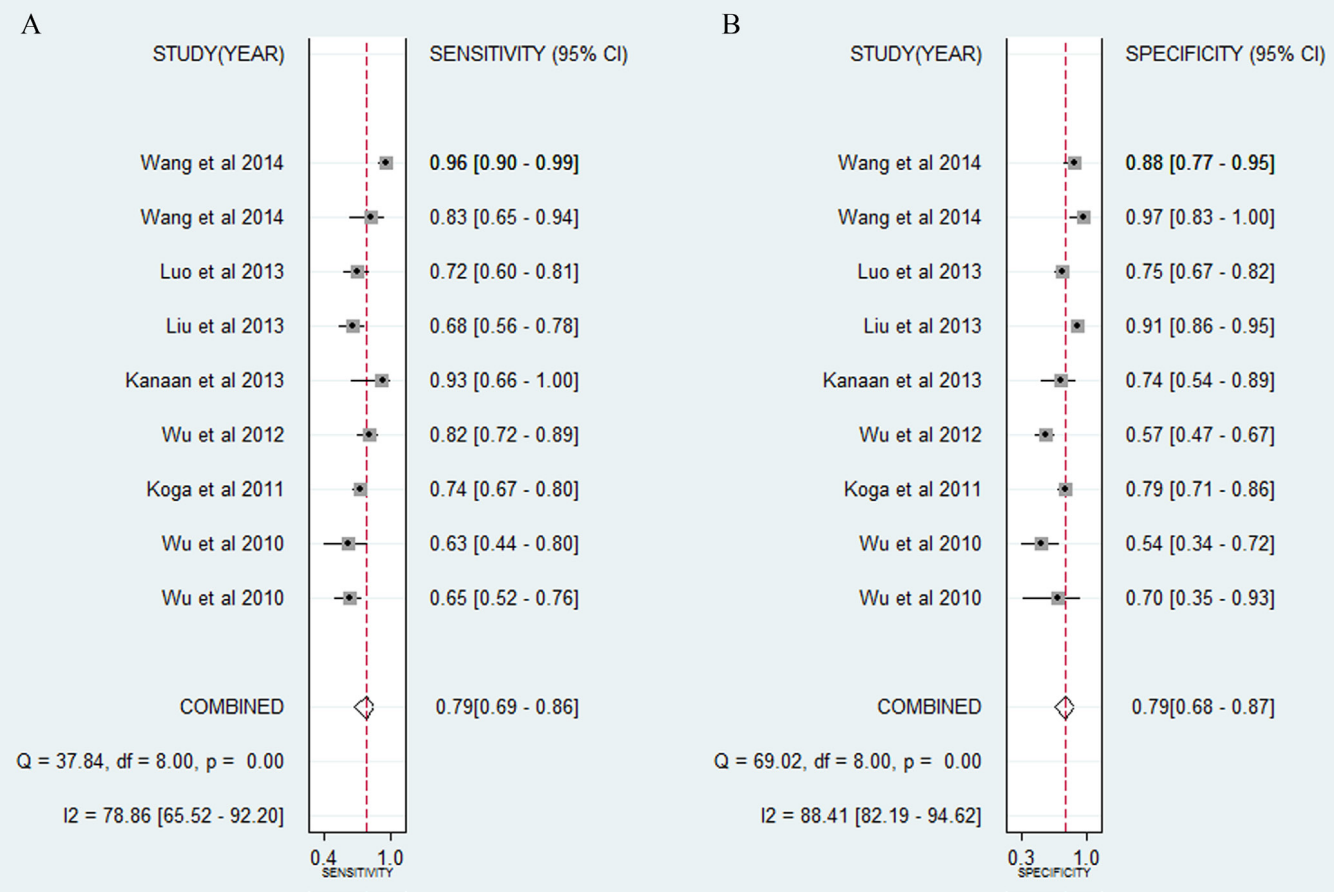
Forest plots of sensitivities and specificities of miR-21-related combination markers in the diagnosis of CRC

**Figure 4:**
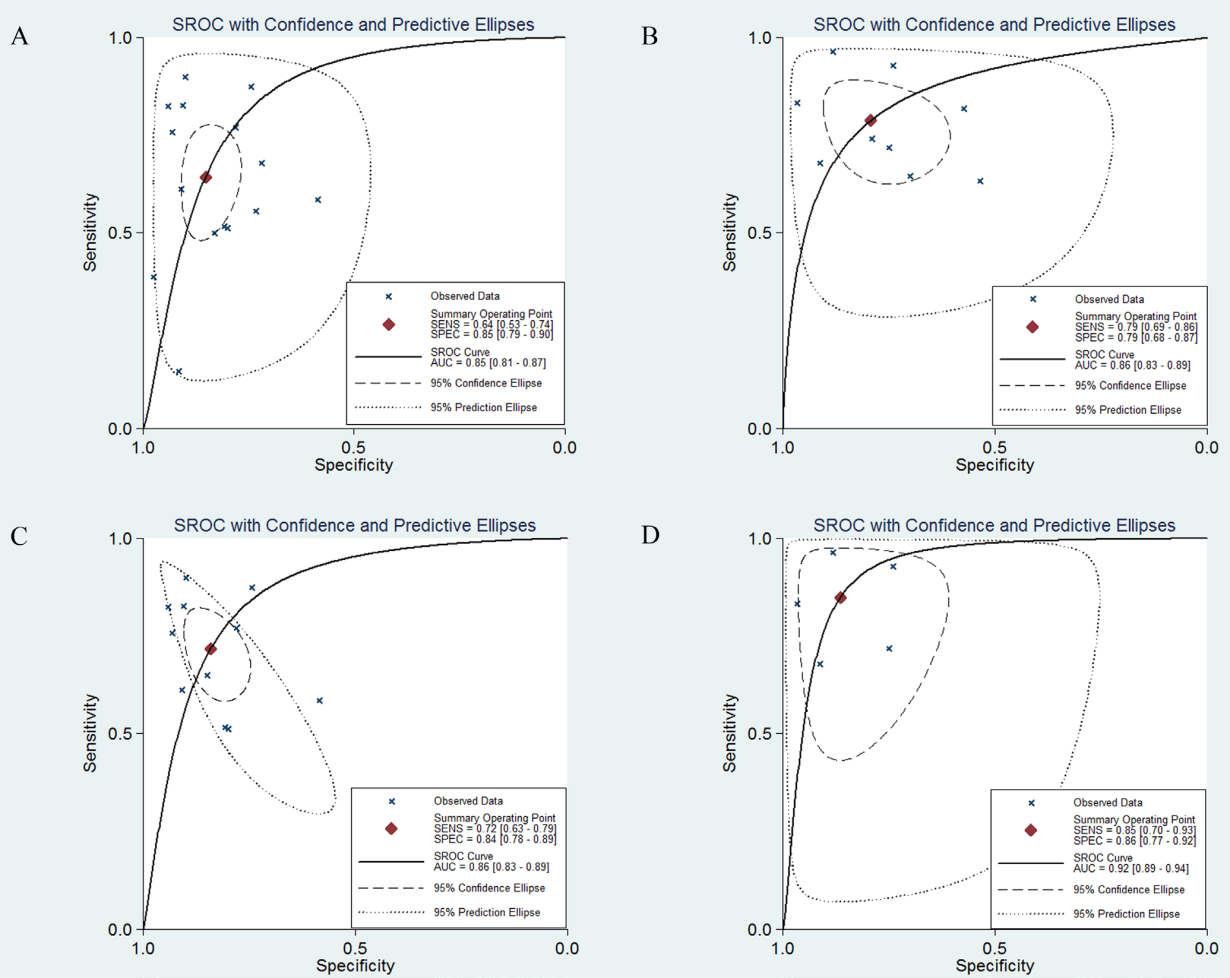
SROC curves in the diagnosis of CRC **A.** SROC curve for miR-21 alone. **B.** SROC curve for miR-21-related combination markers. **C.** SROC curve for miR-21 alone in circulating samples. **D.** SROC curve for miR-21-related combination markers in circulating samples.

The pooled positive likelihood ratio (PLR) and negative likelihood ratio (NLR), which are deemed to be more valuable than sensitivity or specificity for clinical applications, were also calculated, the results for which were 4.33 (3.04-6.17) and 0.42 (0.31-0.57), respectively. The combined PLR indicated that patients with CRC had a nearly four-fold greater chance of having an elevated miR-21 compared with patients without CRC. The pooled NLR meant that the probability of the patient having CRC is 42% if the miR-21 is negative.

#### Threshold effect

The threshold effect has been considered to result from the differences between sensitivity and specificity. In the present study, Spearman's correlation coefficient of sensitivity and specificity was selected as a representative way of exploring the threshold effect. According to the analysis, Spearman's correlation coefficient was 0.068, with a *P* value of 0.803 (*P* > 0.05), suggesting that there was no heterogeneity from the threshold effect.

#### Subgroup and meta-regression analyses

In this study, subgroup analyses were conducted to explore the possible sources of heterogeneity. We found that the pooled sensitivity, specificity, and AUC of the studies were 0.61 (95% CI: 0.46-0.74), 0.87 (0.81-0.91), and 0.85 (0.82-0.88) for Asian populations versus 0.71 (0.54-0.84), 0.82 (0.69-0.90), and 0.84 (0.80-0.87) for Caucasian sones.

Subgroup analysis by sample source revealed that there was no significant difference in the diagnostic accuracy between studies with plasma and serum, with sensitivity of 0.69 (95% CI: 0.54-0.80) versus 0.75 (0.65-0.83), specificity of 0.86 (0.75-0.92) versus 0.84 (0.78-0.88), and AUC of 0.85 (0.82-0.88) versus 0.87 (0.84-0.90). However, both of these exhibited higher diagnostic accuracy than studies with feces, for which the pooled sensitivity, specificity, and AUC were 0.37 (0.21-0.57), 0.89 (0.76-0.96), and 0.72 (0.68-0.76). With respect to the subgroup analysis, to determine the difference between blood-based and feces-based samples, we found that the diagnostic sensitivity and AUC for circulating samples (Figure 4C) were significantly higher than for fecal samples, while the diagnostic specificity for fecal samples was higher than for circulating ones. Overall, circulating samples were more sensitive than fecal samples for detecting CRC. In particular, serum miR-21 assays exhibited slightly higher overall diagnostic power than plasma miR-21.

Pooled studies with a small sample size exhibited higher diagnostic sensitivity of 0.71 (95% CI: 0.62-0.79) compared with studies with a large sample size, for which the value was 0.51 (0.32-0.70); in contrast, a large sample size was associated with specificity of 0.88 (0.80-0.94) compared with the value for a small sample size of 0.82 (0.74-0.88). The overall predictive accuracy in AUC was found to be similar between small and large sample sizes, for which the values were 0.83 (0.80-0.86) and 0.84 (0.81-0.87).

Meta-regression analysis indicated that sample source (*P* > 0.10), ethnicity (*P* > 0.10), and sample size (*P* > 0.10) did not significantly affect the pooled results. Therefore, meta-regression could not enable us to identify the variable sources that may contribute to the heterogeneity of the diagnostic accuracy among the included studies.

#### Sensitivity analysis and publication bias

Sensitivity analysis identified five studies that deviated from the others [31, 36, 37, 42]. After removing them, the I^2^ values for sensitivity and specificity dropped from 94.58% to 73.20% and from 84.41% to 47.99%, respectively. However, there were no significant changes for the newly pooled results.

To evaluate publication bias, Deeks’ funnel plot was selected. The funnel plots exhibited no symmetry (Figure 6A) for all enrolled studies and Deeks’ test returned a P value of 0.36, revealing no obvious publication bias in this analysis.

**Figure 5:**
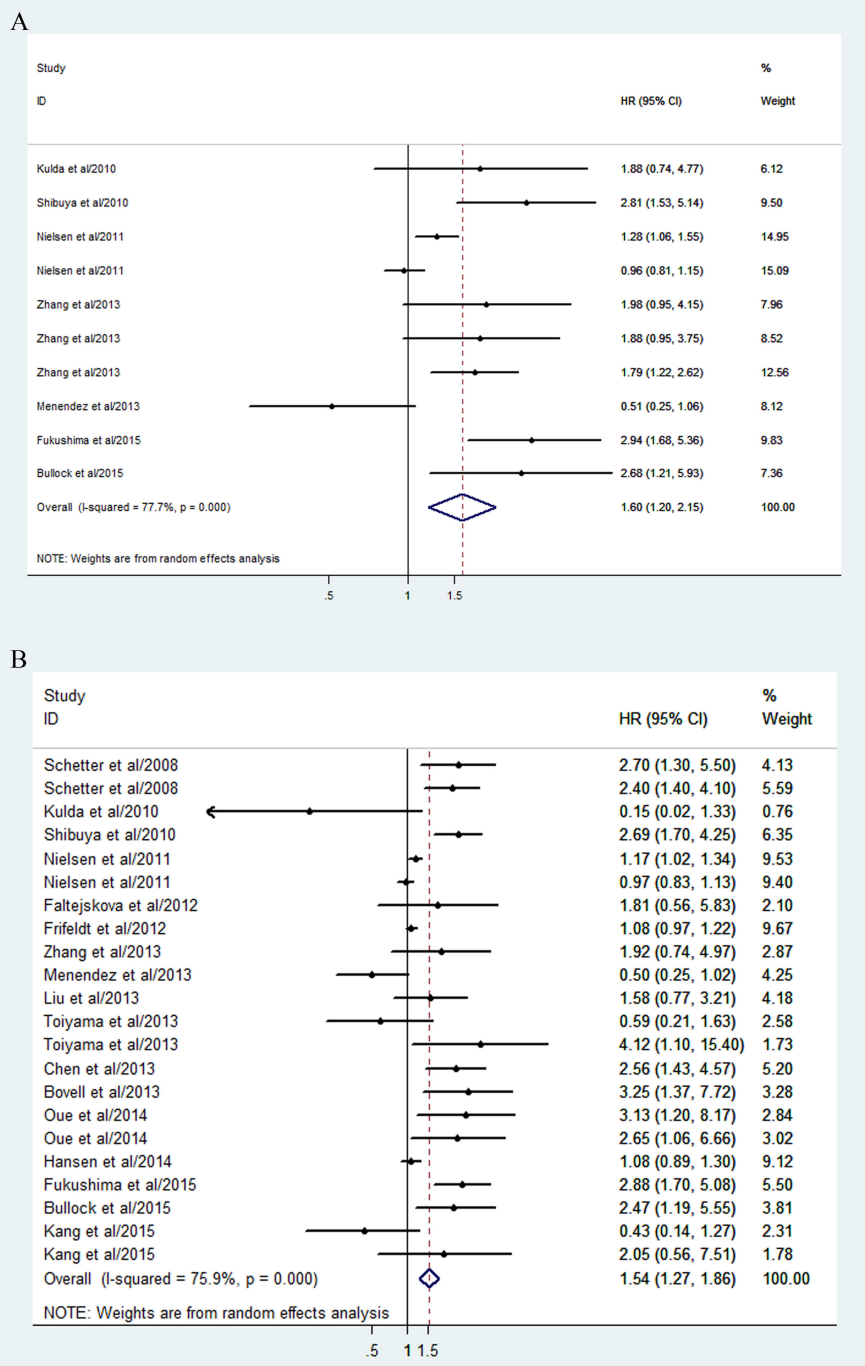
Forest plots of the correlation between miR-21 expression level and CRC prognosis **A.** Forest plot of DFS. **B.** Forest plot of OS.

**Figure 6:**
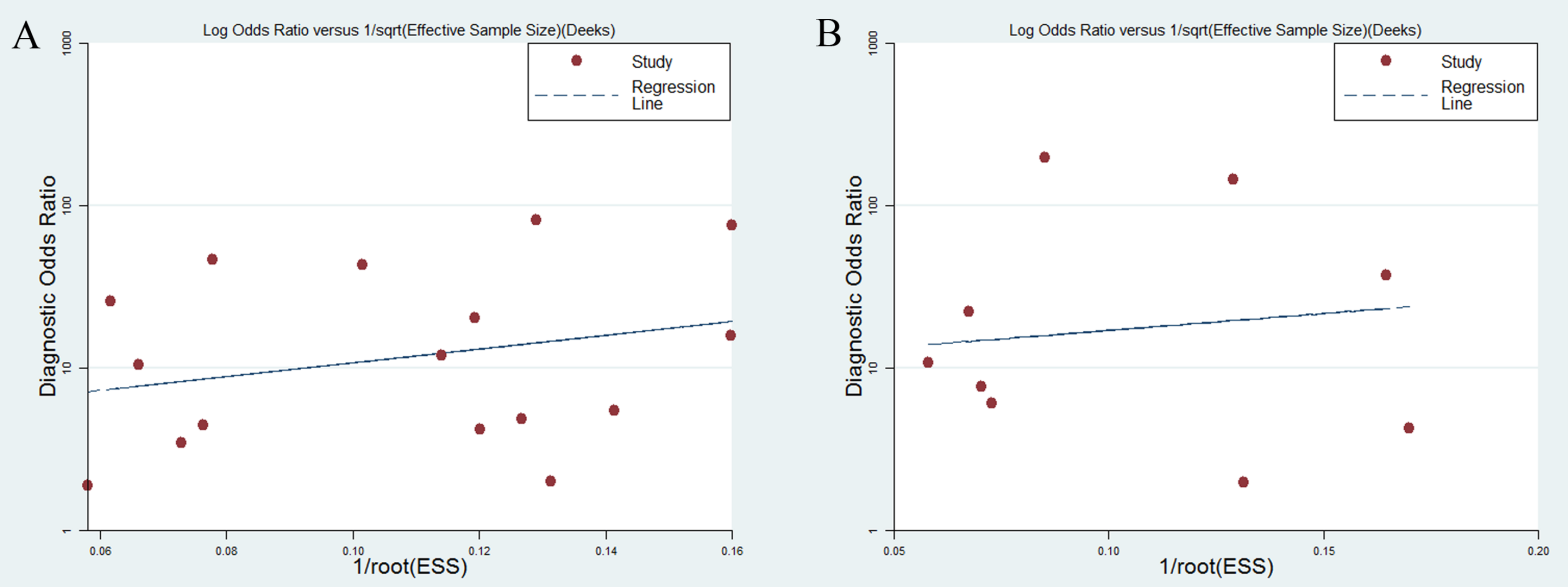
Deeks’ funnel plots for the assessment of potential bias in the meta-analysis for diagnosis **A.** Funnel plot of the studies on miR-21 alone. **B.** Funnel plot of the studies on miR-21-related combination markers.

### Diagnostic meta-analysis of miR-21-related combination markers in CRC

#### Study characteristics and quality assessment

In the nine reported studies on miR-21-related combination markers, a total of 752 patients and 633 normal participants were available for this diagnostic meta-analysis. Table 1B shows the main characteristics of the nine relevant studies. Among these studies, seven were on Asians and two were on Caucasians. Regarding the sample sources, four studies focused on feces, while the remaining five focused on combination markers in blood. All studies measured the expression of combination markers by qRT-PCR. The assessment of the quality of each study indicated that the included studies were suitable for quantitative synthesis.

**Table 1B:** The main features of the included studies on miR-21-related combination markers

Author	Year	Country	Ethnicity	Case/ Control	Sample	miRNA list	AUC	Se	Sp	QUADAS
Wu et al	2010	China	Asian	27/48	Feces	miR-21,miR-92	Na	65.0%	70.0%	3
Wu et al	2010	China	Asian	32/26	Feces	miR-21,miR-92	Na	63.0%	54.0%	3
Koga et al	2010	Japan	Asian	197/119	Feces	miR-21, miR-17-92, miR-135	Na	74.1%	79.0%	3
Wu et al	2012	China	Asian	88/101	Feces	miR-21,miR-92a	Na	81.8%	57.4%	4
Kanaan et al	2012	America	Caucasian	15/26	Plasma	miR-21,miR-331, miR-15b	Na	93.0%	74.0%	4
Liu et al	2013	China	Asian	200/80	Serum	miR-21,miR-92	0.847	68.0%	91.2%	5
Luo et al	2013	Germany	Caucasian	80/144	Plasma	miR panels	0.745	71.8%	75.0%	4
Wang et al	2014	China	Asian	30/30	Serum	miR-21, let-7g, miR-31, miR-92a, miR-181b, miR-203	0.900	83.3%	96.7%	5
Wang et al	2014	China	Asian	83/59	Serum	miR-21, let-7g, miR-31, miR-92a, miR-181b, miR-203	0.923	96.4%	88.1%	5

**Table 1C:** The main features of the included studies for the prognostic meta-analysis

Author	Year	Country	Ethnicity	Type	Sample	N	Age	Stage	Survival results	Follow-up(months)	HR (95% CI)
Kulda et al	2010	Czech	Caucasian	CRC	tissue	46	62.8	I-IV	DFS	45.2	1.88(0.74,4.77)
Shibuya et al	2010	Japan	Asian	CRC	tissue	156	65	I-IV	DFS	44	2.81(1.53,5.14)
Nielsen et al	2011	Denmark	Caucasian	CC	tissue	129	70	II	DFS	≧60	1.28(1.06,1.55)
Nielsen et al	2011	Denmark	Caucasian	RC	tissue	67	70	II	DFS	≧60	0.96(0.81,1.15)
Zhang et al	2013	China	Asian	CC	tissue	138	65	II	DFS	66	1.98(0.95,4.15)
Zhang et al	2013	China	Asian	CC	tissue	137	65	II	DFS	66	1.88(0.95,3.75)
Zhang et al	2013	China	Asian	CC	tissue	460	65	II	DFS	66	1.79(1.22,2.62)
Menendez et al	2013	Spain	Caucasian	CRC	Serum	102	71.6	I-IV	DFS	23	0.51(0.25,1.06)
Fukushima et al	2015	Japan	Asian	CRC	tissue	306	65	I-IV	DFS	48	2.94(1.68,5.36)
Bullock et al	2015	UK	Caucasian	CRC	tissue	50	72	I-IV	DFS	73	2.68(1.21,5.93)
Schetter et al	2008	America	Caucasian	CC	tissue	71	64.4	I-IV	OS	80	2.70(1.30,5.50)
Schetter et al	2008	China	Asian	CC	tissue	103	55.8	I-IV	OS	84.6	2.40(1.40,4.10)
Kulda et al	2010	Czech	Caucasian	CRC	tissue	46	62.8	I-IV	OS	45.2	0.15(0.02,1.33)
Shibuya et al	2010	Japan	Asian	CRC	tissue	156	65	I-IV	OS	44	2.69(1.70,4.25)
Nielsen et al	2011	Denmark	Caucasian	CC	tissue	129	70	II	OS	≧60	1.17(1.02,1.34)
Nielsen et al	2011	Denmark	Caucasian	RC	tissue	67	70	II	OS	≧60	0.97(0.83,1.13)
Faltejskova et al	2012	Czech	Caucasian	CRC	tissue	44	67	I-IV	OS	84	1.81(0.56,5.83)
Frifeldt et al	2012	Denmark	Caucasian	CC	tissue	520	71.9	II	OS	84	1.08(0.97,1.22)
Zhang et al	2013	China	Asian	CRC	tissue	79	62.9	I-IV	OS	65.9	1.92(0.74,4.97)
Menendez et al	2013	Spain	Caucasian	CRC	Serum	102	71.6	I-IV	OS	23	0.50(0.25,1.02)
Liu et al	2013	China	Asian	CRC	Serum	166	57.09	I-IV	OS	36.4	1.58(0.77-3.21)
Toiyama et al	2013	Japan	Asian	CRC	tissue	153	67.5	I-IV	OS	44	0.59(0.21,1.63)
Toiyama et al	2013	Japan	Asian	CRC	Serum	153	67.5	I-IV	OS	44	4.12(1.10,15.4)
Chen et al	2013	China	Asian	CRC	tissue	195	66	I-IV	OS	60	2.56(1.43,4.57)
Bovell et al	2013	America	Caucasian	CRC	tissue	55	65	IV	OS	198	3.25(1.37,7.72)
Oue et al	2014	Japan	Asian	CC	tissue	87	63	II-III	OS	54	3.13(1.20,8.17)
Oue et al	2014	Genmany	Caucasian	CC	tissue	145	70	II	OS	51.6	2.65(1.06,6.66)
Hansen et al	2014	Denmark	Caucasian	CC	tissue	554	74	II-IV	OS	60	1.08(0.89,1.30)
Fukushima et al	2015	Japan	Asian	CRC	tissue	306	65	I-IV	OS	48	2.88(1.70,5.08)
Bullock et al	2015	UK	Caucasian	CRC	tissue	50	72	I-IV	OS	73	2.47(1.19,5.55)
Kang et al	2015	Korea	Asian	CC	tissue	173	63	II-III	OS	80	0.43(0.14,1.27)
Kang et al	2015	Korea	Asian	RC	tissue	104	63	II-III	OS	80	2.05(0.56,7.51)

AUC: area under the curve; Se: sensitivity; Sp: specificity; QUADAS: quality assessment of diagnostic accuracy studies. This scoring system comprises seven questions, requiring an answer of “yes,” “no,” or “unclear.”

An answer of “yes” gets a score of 1, while an answer of “no” or “unclear” gets a score of 0; miR panels: miR-18a, miR-20a, miR-21, miR-29a, miR-92a, miR-106b, miR-133a, miR-143, miR-145, miR-342-3p, miR-532-3p, miR-181b; NA: not available; N: number of participants; DFS: disease-free survival; OS: overall survival; HR: hazard ratio; 95% CI: 95% confidence interval.

#### Diagnostic accuracy of miR-21-related combination markers in CRC

The forest plot (Figure 3) indicated that the overall combined results for sensitivity and specificity were 0.79 (95% CI: 0.69-0.86) and 0.79 (0.68-0.87). Significant heterogeneity was observed since the Q value was 37.84 (*P* < 0.01) and I^2^ was 78.86% (65.52-92.20) for sensitivity, while the Q value was 69.02 (*P* < 0.01) and I^2^ was 88.41% (82.19-94.62) for specificity. Other parameters for all of the results together were also exported: pooled PLR was 3.79 (2.30-6.23), NLR was 0.27 (0.17-0.42), and DOR was 14.15 (5.93-33.76). The AUC (Figure 4B) was 0.86 (0.83-0.89), indicating relatively high predictive power.

**Table 2:** Results of subgroup and meta-regression analyses in the diagnosis meta-analysis

	Subgroup	Number of studies	Se (95% CI)	Sp (95% CI)	AUC (95% CI)	Meta-regression (p-value)
**Individual**	Ethnicity					0.6504
	Caucasian	5	0.71(0.54-0.84)	0.82(0.69-0.90)	0.84(0.80-0.87)	
	Asian	11	0.61(0.46-0.74)	0.87(0.81-0.91)	0.85(0.82-0.88)	
	Sample size					0.6458
	<100	10	0.51(0.32-0.70)	0.88(0.80-0.94)	0.83(0.80-0.86)	
	>100	6	0.71(0.62-0.79)	0.82(0.74-0.88)	0.84(0.81-0.87)	
	Sample type 1					0.1730
	Plasma	6	0.69(0.54-0.80)	0.86(0.75-0.92)	0.85(0.82-0.88)	
	Serum	5	0.75(0.65-0.83)	0.84(0.78-0.88)	0.87(0.84-0.90)	
	Feces	4	0.37(0.21-0.57)	0.89(0.76-0.96)	0.72(0.66-0.76)	
	Tissue	1	0.68	0.72	0.746	
	Sample type 2					
	Circulation	11	0.72(0.63-0.79)	0.84(0.78-0.89)	0.86(0.83-0.89)	
	Feces	4	0.37(0.21-0.57)	0.89(0.76-0.96)	0.72(0.66-0.76)	
	Tissue	1	0.68	0.72	0.746	
**Combination**	Sample type					0.0119
	Circulation	5	0.85(0.70-0.93)	0.86(0.77-0.92)	0.92(0.89-0.94)	
	Feces	4	0.73(0.65-0.80)	0.66(0.53-0.78)	0.76(0.72-0.80)	
	Sample size					0.8999
	<100	4	0.73(0.60-0.83)	0.77(0.53-0.91)	0.80(0.76-0.83)	
	>100	5	0.81(0.67-0.89)	0.80(0.68-0.89)	0.87(0.84-0.90)	
	Ethnicity					0.0147
	Caucasian	2	--	--	--	
	Asian	7	0.78(0.67-0.87)	0.81(0.66-0.90)	0.86(0.83-0.89)	
	Number of miRNAs					0.0437
	two	4	0.71(0.63-0.78)	0.73(0.51-0.87)	0.76(0.72-0.79)	
	two more	5	0.85(0.72-0.92)	0.83(0.75-0.88)	0.89(0.86-0.92)	

**Table 3:** Results of subgroup and meta-regression analyses in the prognostic meta-analysis

Outcome	Subgroup	Number of studies	HR (95% CI)	Heterogeneity (I^2^)	P_heterogeneity_	Meta-regression (p-value)
**DFS**	Ethnicity					0.048
	Caucasian	5	1.16(0.84-1.62)	73.7%	0.004	
	Asian	5	1.60(0.68-2.74)	0.0%	0.567	
	Sample type					0.017
	Blood	1	0.51(0.25-1.05)	--	--	
	Tissue	9	1.76(1.31-2.36)	76.6%	0.001	
	Cancer type					0.012
	CRC	5	1.86(0.96-3.60)	76.7%	0.002	
	RC	1	0.96(0.68-2.74)	--	--	
	CC	4	1.50(1.20-1.89)	24.6%	0.264	
	Multivariate analyses					
	Yes	3	1.65(0.58-4.71)	87.9%	<0.001	
	No	7	1.49(1.14-1.96)	68.5%	0.004	
**OS**	Ethnicity					0.144
	Caucasian	11	1.20(1.00-1.43)	69.0%	0.001	
	Asian	11	2.02(1.47-2.79)	75.9%	0.039	
	Sample type					0.179
	Blood	3	1.34(0.45-4.01)	79.3%	0.008	
	Tissue	19	1.58(1.30-1.93)	76.7%	0.001	
	Cancer type					0.269
	CRC	12	1.76(1.18-2.63)	67.0%	0.001	
	RC	2	1.06(0.66-1.68)	20.6%	0.262	
	CC	8	1.33(1.08-1.64)	71.6%	0.001	
	Multivariate analyses					
	Yes	15	1.82(1.28-2.59)	79.7%	<0.001	
	No	7	1.13(0.95-1.35)	53.0%	0.047	

#### Threshold effect

Spearman's rank correlation was also studied. The results identified no heterogeneity resulting from the threshold effect, from a Spearman's correlation coefficient of −0.433 with *P* = 0.244.

#### Subgroup and meta-regression analyses

Pooled studies on a large sample size exhibited higher diagnostic accuracy than studies on a small sample size, with sensitivity of 0.81 (95% CI: 0.67-0.89) versus 0.73 (0.60-0.83), specificity of 0.80 (0.68-0.89) versus 0.77 (0.53-0.91), and AUC of 0.87 (0.84-0.90) versus 0.80 (0.76-0.83). Subgroup analysis by the number of biomarkers suggested that two more combination markers offered more powerful diagnostic value of CRC than two combination markers, with sensitivity of 0.85 (95% CI: 0.72-0.92) versus 0.71 (0.63-0.78), specificity of 0.83 (0.75-0.88) versus 0.73 (0.51-0.87), and AUC of 0.89 (0.86-0.92) versus 0.76 (0.72-0.79). Furthermore, circulating miRNA combination markers (Figure 4D) had a higher level of predictive power than combination markers in feces, with sensitivity of 0.85 (95% CI: 0.70-0.93) versus 0.73 (0.65-0.80), specificity of 0.86 (0.77-0.92) versus 0.66 (0.53-0.78), and AUC of 0.92 (0.89-0.94) versus 0.76 (0.72-0.80). Among the nine studies, seven studies detected the miR-21 expression in Asian populations. Hence, subgroup analysis was also performed by Asian populations. The pooled sensitivity, specificity, and AUC were 0.78 (95% CI: 0.67-0.87), 0.81 (0.66-0.90), and 0.86 (0.83-0.89).

As suggested by Table 2, the results of the meta-regression were consistent with the conclusion provided by the subgroup analysis. The meta-regression analysis suggested that sample source (*P* < 0.05), ethnicity (*P* < 0.05), and number of combination markers (*P* < 0.05) might be variable sources of heterogeneity in the diagnostic accuracy across the eligible studies.

#### Sensitivity analysis and publication bias

We conducted a sensitivity analysis, but failed to determine the sources of heterogeneity from the results. Deeks’ test returned a *P* value of 0.77, suggesting a low likelihood of publication bias in the diagnostic meta-analysis for miR-21-related combination markers (Figure 6B).

### Prognostic meta-analysis

#### Study characteristics and quality assessment

A total of 1591 participants from 10 studies and 3458 participants from 22 studies were included in the study evaluating OS and PFS, respectively. The main characteristics of the included studies are presented in Table 1C. These eligible studies all involved a retrospective design and focused on patients from nine countries. The ethnic backgrounds of the patients were classified as Asian or Caucasian. Tissue samples were used in 28 studies, while 4 studies used serum samples. Most studies investigated miR-21 by qRT-PCR. The results of quality assessment are listed in Tables S1A and S1B.

#### Correlation between miR-21 expression and DFS

For studies evaluating DFS, clear heterogeneity was found among the studies on miR-21 (*P* < 0.05, I^2^ = 77.7%). Consequently, we calculated the combined HR and the corresponding 95% CI based on a random model, which was 1.60 (95% CI: 1.20-2.15, *P* < 0.01) for all of the studies (Figure 5A), indicating that elevated miR-21 expression predicted a shorter DFS for patients with CRC.

Then, a meta-regression analysis was conducted to reveal the source of the heterogeneity. It revealed that ethnicity, sample source, and cancer type may have contributed to the heterogeneity.

Interestingly, when it came to the subgroup analysis by ethnicity, it was revealed that the combined HR of DFS was 2.15 (95% CI: 1.68-2.74, *P* < 0.01) in Asian populations with CRC versus 1.16 (0.84-1.62, *P* = 0.37) in Caucasians. Among the ten studies, nine explored the correlation between tissue miR-21 and prognosis. Hence, we also analyzed the studies by tissue. It was concluded that a high level of tissue miR-21 indicated a shorter DFS with combined HR of 1.76 (1.31-2.36, *P* < 0.01) for CRC patients. In addition, the combined HR was 1.86 (0.96-3.60, P = 0.06) in CRC patients and 1.50 (1.20-1.89, *P* < 0.01) in patients with colon cancer (CC).

In the sensitivity analysis, after excluding the studies by Nielsen et al. [47] and Menendez et al. [49], the heterogeneity was clearly reduced (I^2^ = 54.6%, *P* = 0.031) and the newly derived combined HR was 1.95 (1.48-2.58, *P* < 0.01).

#### Correlation between miR-21 expression and OS

For studies evaluating OS, a random model was selected as well due to the heterogeneity across studies (*P* < 0.05, I^2^ = 75.9%). It indicated that higher miR-21 expression predicted shorter OS in CRC (HR: 1.54; 95% CI, 1.27-1.86, *P* < 0.01) (Figure 5B).

The meta-regression revealed no significant results on the source of the heterogeneity between studies, with respect to ethnicity, sample source, and cancer type. In the subgroup analysis, it was concluded that the combined HR for OS was 2.02 (95% CI: 1.47-2.79, *P* < 0.01) in Asians versus 1.20 (1.00-1.43, *P* = 0.05) in Caucasians. As regards sample source, the combined HR for OS was 1.58 (1.30-1.93, *P* = 0.61) in tissue sample and 1.34 (0.45-4.01, *P* < 0.01) in serum. When we grouped the meta-analysis by cancer type, we found that the combined HR for OS was 1.33 (1.08-1.64, *p* = 0.007) in colon cancer, 1.76 (1.18-2.63, *p* = 0.006) in CRC, and 1.06 (0.66-1.68, *p* = 0.818) in rectal cancer (RC).

Sensitivity analysis was also conducted, from the results of which we found that, when five studies were discarded [46, 50, 52, 55, 56], the outcome of the sensitivity analysis was more stable (I^2^ dropped from 75.9% to 61.9%, *P* < 0.01).

#### Publication bias

**Figure 7:**
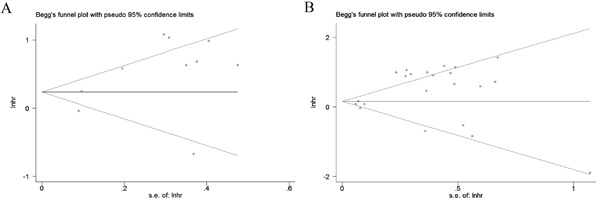
Begg's funnel plots for the assessment of publication bias in the meta-analysis for prognosis **A**. Funnel plot of the studies for DFS. **B**. Funnel plot of the studies for OS.

Finally, Begg's funnel plot and Egger's test were applied to assess publication bias (Figures 7A and 7B). The P values of Egger's regression intercept for DFS and OS were 0.069 and 0.02, respectively, suggesting no obvious publication bias in the quantitative synthesis for evaluating DFS, while publication bias did exist in the meta-analysis for assessing OS.

## DISCUSSION

Tumor biomarkers are critical for diagnosing cancer, predicting its outcome, and selecting an appropriate therapeutic method [60]. Biomarkers that can not only play important roles in early detection of CRC but also predict patients’ outcome should be a greater focus of attention in clinical research [61]. Given their involvement in various important biological processes, including cell growth, differentiation, apoptosis, cancer development, and metastasis, miRNAs may be considered perfect diagnostic, therapeutic, and prognostic biomarkers for CRC [9]. As one of the most researched miRNAs, miR-21 has a high degree of stability and thus has great potential as a biomarker for CRC [62]. Nevertheless, a series of quantitative analyses were conducted to investigate its diagnostic or prognostic value, which generated some conflicting results across the studies. The inconsistent findings prompted us to carry out this comprehensive and up-to-date research, to investigate the diverse values of miR-21 in a clinical context, including its diagnostic and prognostic abilities in CRC.

The present meta-analysis for individual miR-21 in CRC indicated that miR-21 presented diagnostic sensitivity of 64%, specificity of 85%, and AUC of 0.85. These three representative parameters confirmed the accuracy of miR-21 as a promising noninvasive predictor for examining CRC. Nevertheless, the values for PLR (4.33) and NLR (0.42) in this study suggested caution regarding the diagnostic power of miR-21 alone for screening CRC patients, as PLR > 10 and NLR < 0.1 are the thresholds representing high accuracy. Significant heterogeneity was discovered for all parameters of diagnosis, including sensitivity, specificity, PLR, NLR, and DOR. The threshold effect, subgroup, meta-regression, and sensitivity analyses are necessary approaches to explore potential heterogeneity. Given Spearman's correlation coefficient of 0.068 with a *P* value of 0.803 (*P* > 0.05), no heterogeneity due to the threshold effect was confirmed. Meanwhile, meta-regression failed to identify potential sources that may contribute to the heterogeneity of the diagnostic accuracy among the included studies. With regard to the subgroup analysis, we concluded that miR-21 in Asian populations has similar diagnostic accuracy to miR-21 in Caucasian ones. In addition, the specimen type may influence the diagnostic accuracy. Specifically, it was revealed that blood-based (plasma or serum) assays had significantly better overall diagnostic accuracy than feces-based ones, although feces-based assays exhibited a high level of diagnostic specificity. Importantly, circulating miR-21 was considered to be more suitable for detecting CRC, given its abundance and the stability of its expression in circulating samples during the diagnosing process [63]. In addition, consistent with previous meta-analyses [20], serum miR-21 exhibited stronger performance for distinguishing CRC patients from healthy people compared with that in plasma, given values of sensitivity of 0.75 versus 0.69, specificity of 0.84 versus 0.86, and AUC of 0.87 versus 0.85. Thus, we recommended serum as the most suitable specimen type in the following diagnostic studies.

From the perspective of cancer evolution, a single biomarker is unlikely to dictate the complicated evolutionary process at the systemic level. Based on the hypothesis that combination biomarkers will help to explain the internal mechanisms of CRC as well as the external factors influencing it, we carried out a meta-analysis for miR-21-related combination markers in CRC to check whether they were better than miR-21 alone in diagnosing CRC. The pooled results for sensitivity, specificity, PLR, NLR, DOR, and AUC were 0.79, 0.79, 3.79, 0.27, 14.15, and 0.86, suggesting that the diagnostic performance was relatively high. According to the results, a large sample size, two more combination markers, and blood-based miRNA assays exhibited higher diagnostic power than a small sample size, two combination markers, and feces-based miRNA assays, respectively. When compared with the results for miR-21 alone among the different sample sources, circulating miR-21-related combination markers exhibited a higher level of predictive power compared with circulating individual miR-21 assays, with sensitivity of 0.85 versus 0.72, specificity of 0.86 versus 0.84, and AUC of 0.92 versus 0.86. In conclusion, combination markers exhibited high diagnostic value and could be applied in a clinical context, overcoming the insufficient power of a single marker.

The prognostic meta-analyses suggested that miR-21 expression level is a potential marker for predicting survival outcomes in CRC patients. Our results showed that patients with an elevated level of miR-21 may be associated with an increased risk of poor survival, which was 1.60-fold higher for DFS and 1.54-fold higher for OS upon comparison with patients with low miR-21 expression. In particular, the predictive efficacy for DFS and OS was more significant in Asians than in Caucasians. In addition, tissue miR-21 was widely applied to predict the survival outcome in CRC and the results proved the practicability of it as a suitable prognostic biomarker. Meanwhile, circulating miR-21 was also identified as a noninvasive prognostic marker for CRC, although it showed a lower risk of poor survival compared with tissue miR-21. However, significant heterogeneity remained in the meta-analyses of the data for DFS, which can be explained by ethnicity, sample source, and cancer type in the meta-regression. In contrast, this study failed to reveal the factors behind the heterogeneity among the studies for OS. Sensitivity analyses succeeded in identifying several outlier studies, in the analyses of both DFS and OS. Nonetheless, we believe that miR-21 may be a useful biomarker associated with survival outcome.

Although we conducted subgroup, meta-regression, and sensitivity analyses, the heterogeneity in our study was still not fully explained. The heterogeneity across studies was probably due to different baseline characteristics with regard to the distributions of age and gender, histological type, tumor stage, detection approach, and follow-up period. A lack of standardized reporting made it difficult to extract the detailed information about baseline characteristics. In addition, different cut-off points may have contributed to the potential heterogeneity to a certain degree. Furthermore, in the multivariate analysis of the clinicopathological factors for survival, the diversification of adjusted factors in each group may contribute to inaccuracies associated with HR estimation.

Recently, accumulating research has revealed miR-21 to be a promising predictor for CRC detection and prognosis. Our results in this study also supported this conclusion. However, there are still some factors that restrict its application to clinical practice. First, an appropriate standard cut-off value for miR-21 expression is required for the accurate determination of diagnosis and prognosis. The lack of a clear cut-off and the variety of selected cut-off points in different studies are considered to be potential sources of heterogeneity [64]. Second, a consensus should be reached about the standard detection method in terms of the extraction of total RNA and the selection of internal reference RNA for normalization. Although qRT-PCR was the most widely selected method for measuring miR-21 expression, in situ hybridization (ISH) was also used in some studies. Different laboratories also used different internal reference RNA. There is thus a need for further studies on this issue in order to reach agreement on the procedure used for normalization. Third, there is the question of which sample type (plasma, serum, feces, or tissue) should be used. In our study, it was revealed that circulating (especially serum) miR-21 may be a promising marker for detecting CRC, while tissue miR-21 may serve as a useful marker for predicting the survival outcome. Meanwhile, we supposed that tissue miR-21 could predict the overall survival and circulating miR-21 may act as an auxiliary marker, monitoring the level of miR-21 in the body. Besides that, ethnic differences must be considered as the clinical value of miR-21 may vary with patient ethnicity [56]. From our results, we also realized the difference. Another question is as follows: Which has greater clinical utility, single miR-21 or a combination of miR-21 with other miRNAs? According to our findings, miR-21 was significant but not strong enough to discriminate CRC patients from normal controls, while miR-21-related combination markers improved the diagnostic accuracy in blood-based samples. In addition, two more combination markers exhibited higher diagnostic accuracy than two combination markers. Thus, it still remains an open question which and how many miRNAs should be combined with miR-21 for improving the diagnostic power. Finally, researchers have considered using combination markers instead of a single miRNA to increase the power for predicting prognosis. For example, Zhang et al. [48] used a six-miRNA signature including miR-21 in CRC patients, which was shown to be an effective prognostic tool for survival compared with miR-21 alone. Similar conclusions were drawn by Bullock et al. [51]. However, we failed to collect a sufficient number of studies to evaluate the combination biomarkers for their correlations with survival. More high-quality research on this issue is now urgently needed.

Our study has several important strengths compared with the previous meta-analyses. First, we conducted more comprehensive research for estimating the diverse roles of miR-21 in CRC patients. Both diagnostic and prognostic meta-analyses were performed based on sufficient numbers of publications. Next, the clinical values of different sample sources, namely, plasma, serum, feces, and tissue, were investigated, with the aim of identifying the most suitable one for clinical application. In addition, considering the differences in the selected clinical end point in different observational cohorts, the prognostic value of miR-21 for both OS and DFS was evaluated. Moreover, we discussed the diagnostic value of miR-21-related combination markers for the first time. It was revealed that the combination of miR-21 with other miRNAs improved the diagnostic power, which may provide a new path for progress in a clinical context. Finally, several interesting results arose from our meta-analysis, which established a foundation for future observational cohorts and clinical trials. Taking these findings together, although several meta-analyses have already identified miR-21 as a predictor for CRC diagnosis or prognosis, our integrative study is the most accurate and comprehensive one yet.

However, there were also several limitations in our work. First, most of the publications in the diagnostic meta-analyses included healthy participants as controls and were not blind in design. This form of design limits the diagnostic performance. Second, we analyzed data from published studies instead of individual patient data (IPD), which placed restrictions on analyzing all the data in a consistent manner [65]. Third, in the meta-analysis of combination markers, we failed to investigate each component in the combination regarding its diagnostic accuracy with CRC independently, which may generate potential heterogeneity when combining them. Moreover, the sample size for the meta-analysis of combination markers was small and the conclusion thus needs further validation. In addition, only Asians and Caucasians were included in the analyses, so no African populations were enrolled. Furthermore, unpublished studies would likely include increased proportions of negative results, but by definition we were not aware of them and could not include them here. Moreover, we did not extend the search to non-English publications, which could also result in bias as positive results tend to be accepted by English-language journals more easily. Finally, clear publication bias was found in the studies for OS, while Egger's regression intercept in DFS returned a P value of 0.069, showing a tendency for statistical significance.

In conclusion, this meta-analysis comprehensively investigated the application of miR-21 for determining the diagnosis and prognosis of patients with CRC. This study revealed that circulating miR-21 has promise as a predictor for detecting CRC, while tissue miR-21 is a useful marker for predicting CRC survival. Combination miRNA biomarkers have also emerged as a new alternative for clinical application. Nonetheless, further large-scale prospective studies are warranted to develop integrative diagnostic and prognostic models with more appropriate and better prediction capacity.

## MATERIALS AND METHODS

This study was carried out and reported on the basis of the standards formulated in Preferred Reporting Items for Systematic Reviews and Meta-analyses (PRISMA) [66].

### Literature search strategy

Articles published up to May 29, 2016, which were associated with the diagnostic or prognostic application of miR-21 for CRC, were searched based on PubMed, Embase, Cochrane Library, and Web of Science. The search terms used for literature retrieval were as follows: (“colorectal cancer” OR “colorectal tumor’’ OR “colorectal carcinoma” OR “CRC” OR “rectal cancer” OR “rectal tumor” OR “rectal carcinoma” OR “colon cancer” OR “colon tumor” OR “colon carcinoma”) AND (“microRNA-21” OR “miR-21” OR “miRNA-21”). In addition, we also examined the reference lists in identified articles to find any additional relevant studies. Two investigators (Peng and Zhang) independently carried out the literature search and the following tasks.

### Eligibility criteria

The main criteria considered for the enrollment of studies were as follows: (1) they reported research on patients with CRC; (2) they detected miR-21 expression in plasma, serum, feces, or tissues; (3) they made a definitive diagnosis of CRC with the gold standard; (4) they undertook a thorough inquiry into the relationship between miR-21 and CRC detection or DFS or OS; and (5) they provided sufficient data for calculating the rates of true positive (TP), false positive (FP), false negative (FN), and true negative (TN) for diagnostic meta-analysis or HR for prognostic meta-analysis.

Studies were excluded if they were (1) not relevant to our study topic; (2) published in the form of letters, reviews, editorials, or case reports; (3) duplicate publications; (4) non-English publications; or (5) involved unqualified data.

### Data extraction

Two reviewers (Peng and Zhang) independently collected the relevant data from the articles based on standardized forms. Any disagreement on whether a particular study should be included was settled by consulting with a third reviewer (Min) and then reaching a consensus. The following information from the diagnostic and prognostic studies was extracted: name of the first author; time of publication; country of research; ethnicity of research population; number of participants; source of samples; and diagnostic results including sensitivity, specificity, TP, FP, FN, and TN; or prognostic results including follow-up time and HR estimates with 95% CIs for DFS or OS. If HRs or their corresponding 95% CIs for DFS or OS were not directly given in the included articles, they were extracted using Kaplan-Meier survival curves by a method previously introduced by Tierney et al. [67].

### Quality assessment

Quality Assessment of Diagnostic Accuracy Studies 2 (QUADAS-2) was applied to judge the quality of selected publications enrolled in the diagnostic study [68]. For prognostic studies, methodological quality was assessed by following the guidelines of the Newcastle-Ottawa Scale [69].

### Statistical analysis

For the diagnostic meta-analyses, the numbers of patients with TP, FP, FN, and TN test results were retrieved directly or via recalculation based on the combination of the reported diagnostic estimates and the sample size in the included study. All of the pooled parameters were estimated by the bivariate meta-analysis model [70], including (1) sensitivity, (2) specificity, (3) positive likelihood ratio (PLR), (4) negative likelihood ratio (NLR), and (5) diagnostic odds ratio (DOR). The summary receiver operator characteristic (SROC) curve was established based on the sensitivity and specificity of every study [71]. In addition, we calculated the corresponding area under the SROC curve (AUC), which is commonly applied for quantifying the diagnostic power. Furthermore, heterogeneity across studies was assessed using the I^2^ statistic [72]. Possible sources of heterogeneity were explored by conducting subgroup, meta-regression, and sensitivity analyses [73]. Finally, Deeks’ funnel plot was adopted to evaluate the publication bias of the included studies; *P* < 0.05 indicates significant publication bias [74].

With respect to the prognostic meta-analyses, all of the HRs and their 95% CIs were combined to calculate the pooled impact of miR-21 expression on the survival of CRC patients. Cochran's Q test (significant at *P* < 0.05) and I^2^ statistics (ranging from 0% to 100%) were used to check the heterogeneity of the pooled results [75]. A fixed-effect model was selected when homogeneity was fine (*P* > 0.05, I^2^ < 50%); otherwise, a random-effect model was used [76]. Meta-regression, subgroup, and sensitivity analyses were carried out to identify the potential sources of heterogeneity. Finally, Begg's funnel plots were selected to evaluate the included studies for the possibility of publication bias [77].

The statistical analyses were completed using STATA (version 12.0) and Meta-DiSc statistical software (version 1.4) [78]. Values of *P* < 0.05 were deemed to represent statistical significance.

## SUPPLEMENTARY MATERIALS TABLE


