# Supplementary material for: The clinical role of microRNA-21 as a promising biomarker in the diagnosis and prognosis of colorectal cancer: a systematic review and meta-analysis
Source: Oncotarget. 2017 Mar 22;8(27):44893–909. doi: 10.18632/oncotarget.16488 (PMC5546529; doi:10.18632/oncotarget.16488)
Supplement: Supplementary file 1 [file oncotarget-08-44893-s001.docx]

The clinical role of microRNA-21 as a promising biomarker in the diagnosis and prognosis of colorectal cancer: a systematic review and meta-analysis

**Supplementary Material**

**Table S1A The quality assessments for the studies of DFS**

| **Author** | **Representativeness of the exposed cohort** | **Selection of the non exposed cohort** | **Ascertainment of exposure** | **Demonstration that outcome of interest was not present at the start of study** | **Comparability of cohorts on the basis of the design or analysis** | **Assessment of outcome** | **Was follow-up long enough for outcomes to occur** | **Adequacy of follow-up of cohorts** | **Scores** |
| --- | --- | --- | --- | --- | --- | --- | --- | --- | --- |
| Kulda | ✵ | ✵ | ✵ | ✵ | x | ✵ | ✵ | ✵ | 7 |
| Shibuya | ✵ | ✵ | ✵ | ✵ | ✵✵ | ✵ | ✵ | ✵ | 9 |
| Nielsen | ✵ | ✵ | ✵ | ✵ | ✵✵ | ✵ | ✵ | ✵ | 9 |
| Nielsen | ✵ | ✵ | ✵ | ✵ | ✵✵ | ✵ | ✵ | ✵ | 9 |
| Zhang | ✵ | ✵ | ✵ | ✵ | x | ✵ | ✵ | ✵ | 7 |
| Zhang | ✵ | ✵ | ✵ | ✵ | x | ✵ | ✵ | ✵ | 7 |
| Zhang | ✵ | ✵ | ✵ | ✵ | x | ✵ | ✵ | ✵ | 7 |
| Menendez | ✵ | ✵ | ✵ | ✵ | ✵✵ | ✵ | x | ✵ | 8 |
| Fukushima | ✵ | ✵ | ✵ | ✵ | ✵✵ | ✵ | ✵ | ✵ | 9 |
| Bullock | ✵ | ✵ | ✵ | ✵ | ✵✵ | ✵ | ✵ | ✵ | 9 |

**Table S1B The quality assessments for the studies of OS**

| **Author** | **Representativeness of the exposed cohort** | **Selection of the non exposed cohort** | **Ascertainment of exposure** | **Demonstration that outcome of interest was not present at the start of study** | **Comparability of cohorts on the basis of the design or analysis** | **Assessment of outcome** | **Was follow-up long enough for outcomes to occur** | **Adequacy of follow-up of cohorts** | **Scores** |
| --- | --- | --- | --- | --- | --- | --- | --- | --- | --- |
| Schetter | ✵ | ✵ | ✵ | ✵ | ✵✵ | ✵ | ✵ | ✵ | 9 |
| Schetter | ✵ | ✵ | ✵ | ✵ | ✵✵ | ✵ | ✵ | ✵ | 9 |
| Kulda | ✵ | ✵ | ✵ | ✵ | ✵✵ | ✵ | ✵ | ✵ | 9 |
| Shibuya | ✵ | ✵ | ✵ | ✵ | ✵✵ | ✵ | ✵ | ✵ | 9 |
| Nielsen | ✵ | ✵ | ✵ | ✵ | ✵✵ | ✵ | ✵ | ✵ | 9 |
| Nielsen | ✵ | ✵ | ✵ | ✵ | ✵✵ | ✵ | ✵ | ✵ | 9 |
| Faltejskova | ✵ | ✵ | ✵ | ✵ | ✵✵ | ✵ | x | ✵ | 8 |
| Frifeldt | ✵ | ✵ | ✵ | ✵ | ✵✵ | ✵ | ✵ | ✵ | 9 |
| Zhang | ✵ | ✵ | ✵ | ✵ | ✵✵ | ✵ | ✵ | ✵ | 9 |
| Menendez | ✵ | ✵ | ✵ | ✵ | ✵✵ | ✵ | x | ✵ | 8 |
| Liu | ✵ | ✵ | ✵ | ✵ | x | ✵ | x | ✵ | 7 |
| Toiyama | ✵ | ✵ | ✵ | ✵ | ✵✵ | ✵ | ✵ | ✵ | 9 |
| Toiyama | ✵ | ✵ | ✵ | ✵ | ✵✵ | ✵ | ✵ | ✵ | 9 |
| Chen | ✵ | ✵ | ✵ | ✵ | ✵✵ | x | ✵ | ✵ | 8 |
| Bovell | ✵ | ✵ | ✵ | ✵ | ✵✵ | ✵ | ✵ | ✵ | 9 |
| Oue | ✵ | ✵ | ✵ | ✵ | ✵✵ | ✵ | ✵ | ✵ | 9 |
| Oue | ✵ | ✵ | ✵ | ✵ | ✵✵ | ✵ | ✵ | ✵ | 9 |
| Hansen | ✵ | ✵ | ✵ | ✵ | ✵✵ | ✵ | ✵ | ✵ | 9 |
| Fukushima | ✵ | ✵ | ✵ | ✵ | ✵✵ | ✵ | ✵ | ✵ | 9 |
| Bullock | ✵ | ✵ | ✵ | ✵ | ✵✵ | ✵ | ✵ | ✵ | 9 |
| Kang | ✵ | ✵ | ✵ | ✵ | x | ✵ | ✵ | ✵ | 7 |
| Kang | ✵ | ✵ | ✵ | ✵ | x | ✵ | ✵ | ✵ | 7 |

**Notes:** ‘x’ represents no score in corresponding items.
